# The Critical Roles of Proteostasis and Endoplasmic Reticulum Stress in Atrial Fibrillation

**DOI:** 10.3389/fphys.2021.793171

**Published:** 2022-01-04

**Authors:** Padmini Sirish, Daphne A. Diloretto, Phung N. Thai, Nipavan Chiamvimonvat

**Affiliations:** ^1^Division of Cardiovascular Medicine, Department of Internal Medicine, University of California, Davis, Davis, CA, United States; ^2^Department of Veterans Affairs, Northern California Health Care System, Mather, CA, United States; ^3^Department of Pharmacology, University of California, Davis, Davis, CA, United States

**Keywords:** atrial fibrillation, endoplasmic reticulum stress, inflammation, oxidative stress, electrical remodeling, structural remodeling

## Abstract

Atrial fibrillation (AF) remains the most common arrhythmia seen clinically. The incidence of AF is increasing due to the aging population. AF is associated with a significant increase in morbidity and mortality, yet current treatment paradigms have proven largely inadequate. Therefore, there is an urgent need to develop new effective therapeutic strategies for AF. The endoplasmic reticulum (ER) in the heart plays critical roles in the regulation of excitation-contraction coupling and cardiac function. Perturbation in the ER homeostasis due to intrinsic and extrinsic factors, such as inflammation, oxidative stress, and ischemia, leads to ER stress that has been linked to multiple conditions including diabetes mellitus, neurodegeneration, cancer, heart disease, and cardiac arrhythmias. Recent studies have documented the critical roles of ER stress in the pathophysiological basis of AF. Using an animal model of chronic pressure overload, we demonstrate a significant increase in ER stress in atrial tissues. Moreover, we demonstrate that treatment with a small molecule inhibitor to inhibit the soluble epoxide hydrolase enzyme in the arachidonic acid metabolism significantly reduces ER stress as well as atrial electrical and structural remodeling. The current review article will attempt to provide a perspective on our recent understandings and current knowledge gaps on the critical roles of proteostasis and ER stress in AF progression.

## Introduction

Atrial fibrillation (AF) is the most commonly diagnosed sustained arrhythmia, affecting 46 million people worldwide (Kornej et al., 2020). The prevalence of AF has increased 3-fold over the past 50 years due to the aging population (Kornej et al., 2020). AF is associated with a significant increase in morbidity and mortality (Chugh et al., 2001, 2014; Estes et al., 2011), yet, current treatment paradigms have proven largely inadequate (Prystowsky et al., 2010; Wilton et al., 2010; Van Wagoner et al., 2015). Clinically available anticoagulants and anti-arrhythmic drugs aim to prevent clot formation and restore sinus rhythm, respectively; however, they carry the risk of unwanted bleeding and proarrhythmia, including lethal ventricular tachyarrhythmias (Lemme et al., 2018; van Gorp et al., 2020). The success rate for catheter ablation in longstanding persistent AF is only 30–50% (Scherr et al., 2021). Given its rising prevalence and lack of effective treatments, there is a need for a deeper understanding of the mechanisms underlying AF progression.

AF is self-promoting and progressive in nature (Nattel et al., 2020). Ectopic activity represents one of the main mechanism that initiates AF (Staerk et al., 2017). The pulmonary veins (PVs) are the main source of ectopic firing within the atria (Staerk et al., 2017; Nattel et al., 2020). The PVs’ unique electrical properties and complex structure promote ectopic activity and reentry, which is needed to sustain the arrhythmia (Staerk et al., 2017; Nattel et al., 2020). PVs have shorter action potential duration and amplitude compared to atrial cardiomyocytes (CMs; Mahida et al., 2015). Similarly, branched fibers within the PVs have abrupt orientation change, which can potentially allow for reentrant circuits (Nattel et al., 2020). The ectopic firing is mainly due to spontaneous Ca^2+^ leak from the sarcoplasmic reticulum (SR), which in turn activates an inward current *via* the Na^+^-Ca^2+^ exchanger (NCX). This influx of Na^+^ causes the CM to spontaneously depolarize. The ectopic activity from these events directly triggers an AF episode, coupled with conduction abnormalities within the tissue can then generate a reentrant circuit to sustain the arrhythmia.

Aging, as well as injury to the heart, causes the atria to undergo structural remodeling and fibrosis. In response to chronic pressure overload, inflammation or hypoxia, signaling molecules including angiotensin II (ANG II), transforming growth factor-β1 (TGF-β1), platelet-derived growth factor (PDGF), and connective-tissue growth factor (CTGF) activate membrane receptors that lead to the production of extracellular matrix (ECM) and fibrosis (Nattel et al., 2020). Downstream signaling pathways include NLR family pyrin domain containing 3 (NLRP3) and nuclear factor-κB (NF-κB). Conduction slowing from the downregulation of connexins coupled with tissue fibrosis result in the increased propensity for reentrant circuits, which allow for sustained arrhythmias (Nattel et al., 2020).

Given that AF progresses over decades, age is the most significant risk factor (Kornej et al., 2020). Hallmarks of aging, including chronic inflammation and increased reactive oxygen species (ROS) production, damage vasculature and myocardium, ultimately increasing one’s risk of developing AF (Kornej et al., 2020). Hypertension is an underlying condition for a fourth of all AF patients (Kornej et al., 2020). The elevated blood pressure triggers structural remodeling and fibrosis. Similarly, type 2 diabetes mellitus patients have a 40% increased risk of developing AF. Here, oxidative stress and inflammation seen in this condition lead to mitochondrial dysfunction and DNA damage, which can predispose one to AF (Kornej et al., 2020). While AF usually occurs in the presence of other comorbidities, AF can also lead to a variety of complications including heart failure and stroke. Given its clinical significance, the underlying mechanisms of AF must be well understood to design effective therapeutic strategies. The current review article will attempt to provide a perspective on our recent knowledge in the effects of endoplasmic reticulum (ER) stress and inflammation on AF progression.

## Proteostasis

Protein folding is a critical mechanism that sustains specialized cell and tissue function, which ultimately maintains our physiology and protects us from disease (Balch et al., 2008; Powers et al., 2009; Bouchecareilh and Balch, 2011; Hetz et al., 2015). The tightly regulated generation and maintenance of these proteins are known as protein homeostasis or proteostasis (Balch et al., 2008; Powers et al., 2009; Bouchecareilh and Balch, 2011). The ER is the organelle that houses protein synthesis, folding, and maintenance. While about 30% of our proteins are made within the ER, all proteins are transported back to the ER for quality control screenings (Hetz et al., 2015; Navid and Colbert, 2017). Here, over 2,000 chaperones, including heat shock proteins, and degradative molecules make up the proteostasis network (PN), which guides protein synthesis, folding, conformational maintenance, and degradation (Hetz et al., 2015; Navid and Colbert, 2017; Hipp et al., 2019). The chaperones use ATP-dependent and independent interactions to fold proteins into a more energetically favored state and maintain these configuration (Bouchecareilh and Balch, 2011; Hipp et al., 2019). However, if the chaperones are not able to fold the proteins correctly, ER-associated degradation (ERAD) systems, such as the ubiquitin–proteasome system (UPS) and autophagosomal–lysosomal pathway, are able to digest and degrade the proteins to prevent downstream dysfunction (Bouchecareilh and Balch, 2011; Hetz et al., 2015; Navid and Colbert, 2017; Hipp et al., 2019).

PN activity is determined by many signaling pathways, including the unfolded protein response (UPR), heat shock response (HSR), UPS, Ca^2+^ sensing, and inflammatory response (Bouchecareilh and Balch, 2011). Despite being tightly regulated, the PN can become overwhelmed and damaged by various factors, including genetic mutations, pathologies, environmental stressors, and pollutants (Bouchecareilh and Balch, 2011). When the ER’s processing capability cannot meet its demand, the cell begins to experience ER stress (Bouchecareilh and Balch, 2011; Navid and Colbert, 2017; Chadwick and Lajoie, 2019). Here, the ER is overloaded with misfolded proteins, which can become toxic to the cell (Adams et al., 2019).

## ER Stress

Most cells are performing near their functional limits; therefore, overwhelming the ER and triggering ER stress can occur during hypoxia, nutrient deprivation, point mutations that hinder protein folding, redox changes, and Ca^2+^ imbalances that impair PN chaperones and challenge proteostasis (Hetz et al., 2015; Adams et al., 2019). Under ER stress, the cell initiates the UPR, a specialized surveillance system, to prevent damage to the cell (Hetz et al., 2015; Adams et al., 2019). Abnormal proteins are screened before they are secreted out of the ER and potentially hinder cell functions (Hetz et al., 2015). Once ER stress is detected, the UPR initiates signaling cascades that tune protein folding capability and protein synthesis to restore proteostasis (Hetz et al., 2015; Navid and Colbert, 2017; Adams et al., 2019). While the UPR is an effective method for mediating ER stress, if the cell’s condition does not improve, “terminal UPR” will be initiated and ultimately lead to cell apoptosis (Hetz et al., 2015). Given UPR’s lethality if left on too long, chronic ER stress has been identified as a key contributor to many disease pathologies, including neurodegeneration, diabetes, cancer, inflammatory and metabolic disorders, and heart disease (Hetz et al., 2015; Liu and Dudley Jr., 2018; Chadwick and Lajoie, 2019). Therefore, the cell must carefully balance the UPR to safely remedy ER stress and maintain proteostasis.

## UPR Signaling

**Figure 1:**
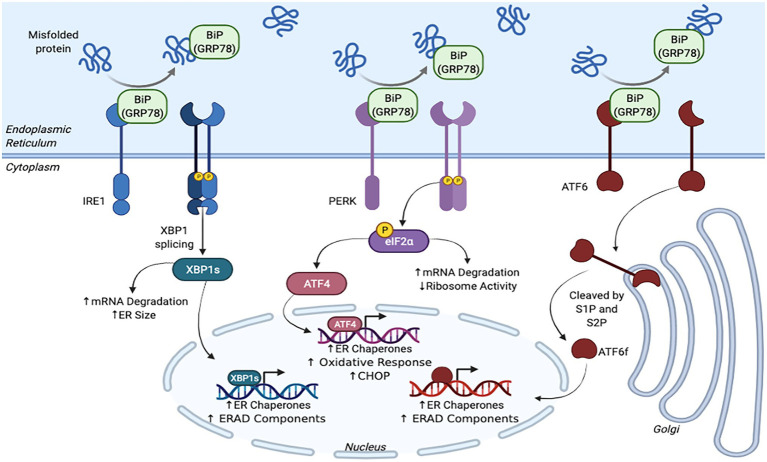
Schematic representation of the unfolded protein response (UPR) pathway.

Early discoveries of an adaptive response to ER stress within yeast *Saccharomyces cerevisiae* prompted the search for a similar mechanism within mammals (Figure 1; Kozutsumi et al., 1988). Soon after, the three main mammalian UPR sensors, inositol requiring enzyme 1α/β (IRE1; Tirasophon et al., 1998), PKR-like ER kinase (PERK; Harding et al., 1999), and activating transcription factor 6α/β (ATF6; Haze et al., 1999) were discovered (Hetz et al., 2015; Adams et al., 2019). The activation of these transmembrane sensors halts *de novo* protein synthesis and increases the ER’s folding capabilities to allow for the proteins within the organelle to be processed effectively and efficiently (Hetz et al., 2015; Adams et al., 2019).

UPR activation is dependent on the chaperone binding immunoglobulin protein/glucose-regulated protein 78 kDa (BiP also known as GRP78) binding to the misfolded proteins within the ER lumen (Hetz et al., 2015; Bhattarai et al., 2020). When inactive, BiP is bound to IRE1, PERK, and ATF6 (Hetz et al., 2015; Chadwick and Lajoie, 2019; Hipp et al., 2019; Bhattarai et al., 2020, 2021). However, once BiP dissociates from these sensors and binds to misfolded proteins, various cascades are initiated that ultimately slow down protein translation, improve folding capabilities, and restore proteostasis.

Once BiP dissociates from IRE1, the sensor is primed for oligomerization and auto-transphosphorylation (Hetz et al., 2015). After undergoing dimerization and phosphorylation, IRE1 undergoes a conformational change that activates its RNase domain (Hetz et al., 2015). The RNase excises an intron within the X-box binding protein 1 (XBP1) mRNA, which shifts the reading frame and activates the transcription factor XBP1s (Hetz et al., 2015; Adams et al., 2019; Bhattarai et al., 2020). This factor translocates to the nucleus and controls protein folding by upregulating chaperones and decreases demand by producing more ERAD components (Navid and Colbert, 2017; Adams et al., 2019; Bhattarai et al., 2020). XBP1s also instructs the ER to grow so that it can accommodate for the excess misfolded proteins (Oakes and Papa, 2015; Liu and Dudley Jr., 2018). In addition, IRE1 activation causes mRNA within the ER to be degraded *via* a process known as regulated IRE1 dependent decay (RIDD; Liu and Dudley Jr., 2018; Bhattarai et al., 2021). Altogether, this response allows for the ER to better manage protein folding and match its proteins synthesis demand (Hetz et al., 2015; Navid and Colbert, 2017; Adams et al., 2019).

The PERK sensor is responsible for regulating protein translation (Adams et al., 2019). Similar to IRE1, once BiP detaches from PERK, the sensor is dimerized and phosphorylated. This activated PERK then phosphorylates eukaryotic initiation factor 2-α-subunit (eIF2α), which inhibits ribosomes, degrades mRNA, and halts global protein synthesis to remedy ER overload (Hetz et al., 2015; Liu and Dudley Jr., 2018; Adams et al., 2019; Bhattarai et al., 2020). Paradoxically, eIF2α also activates activation transcription factor 4 (ATF4), which is involved in antioxidant pathways and increases the ER’s protein folding capabilities by producing more chaperones (Hetz et al., 2015; Liu and Dudley Jr., 2018; Adams et al., 2019; Bhattarai et al., 2020).

The ATF6 sensor, which is regulates protein folding capabilities and ERAD pathways, has a slightly different mechanism of action. When BiP is not bound to this protein, ATF6 displays an export motif. Once it leaves the ER, ATF6 travels to the Golgi complex and becomes activated after being cleaved by site-1 and site-2 proteases (S1P and S2P). ATF6’s cleaved cytosolic domain, ATF6f, localizes within the nucleus and activates transcription factors that upregulate the production of ERAD components as well as protein folding chaperones (Hetz et al., 2015; Adams et al., 2019). Again, these cascades aim to decrease the amount of proteins within the ER and increase its folding capabilities to allow for its quality control machinery to work within its limits.

Together, these three UPR sensors work toward lowering the ER’s protein load and increasing its folding capacity to restore proteostasis (Adams et al., 2019). To match this translational shutdown, ribosomes also dissociate from the ER to prevent further translation (Hetz et al., 2015). Increased ERAD and autophagy activity clears misfolded proteins from the ER to alleviate stress (Hetz et al., 2015). However, if this response is over activated, the cell runs the risk of shifting toward terminal UPR and ultimately becoming apoptotic.

## Chronic ER Stress and Cell Death

Given that one of UPR’s main mechanism of action depends on the halt of new protein translation, if this block was to be prolonged, the cell would not be able to keep up with its metabolic demands and die (Adams et al., 2019). Therefore, the branches of the UPR have the potential to trigger pro-apoptotic cascades if over activated in the case of chronic ER stress and terminal UPR. During prolonged ER stress, IRE1 begins to interact with tumor necrosis factor receptor associated factor-2 (TRAF2) to activate apoptosis signal-regulating kinase 1 (ASK1). This kinase then activates c-Jun amino-terminal kinase (JNK), which ultimately leads to apoptosis (Bhattarai et al., 2020, 2021; Read and Schroder, 2021). The IRE1-TRAF2 interaction is also able to phosphorylate IκB kinase, which in turn causes NF-κB to localize in the nucleus and promote inflammatory gene transcription (Bhattarai et al., 2021). Similarly, if PERK is over activated, the cell can become apoptotic. The downstream transcription factor ATF4 also activates the pro-apoptotic gene CCAAT/enhancer-binding protein homologous protein (CHOP; Hetz et al., 2015; Liu and Dudley Jr., 2018; Bhattarai et al., 2020). Therefore, during chronic ER stress, CHOP is highly expressed and promotes pro-apoptotic pathways. CHOP and ATF4 have also been shown to interact with one another to upregulate protein synthesis, which further burdens the ER (Hetz et al., 2015; Bhattarai et al., 2020, 2021). This in turn depletes ATP and produces ROS, which ultimately leads to cell death (Hetz et al., 2015). This delicate balance between the UPR sensors being protective and promoting cell death highlights the significance of the PN and the need for an effective response to prevent prolonged ER stress and UPR activation.

## ER Stress and Inflammation

ER stress-induced UPR signaling mediates inflammation *via* NF-κB. All three branches of the UPR activate NF-κB *via* gene transcription (Hasnain et al., 2012; Chipurupalli et al., 2021). Under normal conditions, NF-κB forms a complex with inhibitor kappa B (ΙκΒ) and is unable to translocate to the nucleus and activate gene transcription. Under ER stress, IκB is phosphorylated and degraded, ultimately allowing NF-κB to translocate and trigger cytokine expression. Initially, IRE1 branch is activated when the chaperone BiP that is usually bound to the three factors, IRE1, ATF6, and PERK, disengages due to the accumulation of misfolded proteins in the ER. This leads to the autophosphorylation of IRE1 and binding to adaptor protein, TRAF2, activating JNK/AKT pathways (Hasnain et al., 2012; Chipurupalli et al., 2021). The IRE1/TRAF2 complex also activates ΙκΒ kinase, which phosphorylates IκB, leading to NF-κB nuclear translocation and cytokine expression. The autophosphorylation of PERK results in the expression of inflammatory cytokines interleukin (IL)-1, 6, and tumor necrosis factor alpha (TNF-α; Chipurupalli et al., 2021). CHOP which is activated within the PERK branch also regulates NF-κB. Finally, the ATF6 branch of the UPR relies on mTOR/AKT signaling to activate NF-κB, leading to inflammatory cytokine expression.

## Critical Roles of ER Stress in Cardiovascular Disease

As our bodies and cells age, the PN becomes increasingly burdened and is unable to maintain proteostasis (Chadwick and Lajoie, 2019; Hipp et al., 2019). Chaperone expression tends to decline, ultimately allowing for misfolded proteins to accumulate within the ER (Chadwick and Lajoie, 2019; Hipp et al., 2019). Exposure to oxidation further impairs the existing chaperones and contributes to the aggregation of misfolded proteins (Chadwick and Lajoie, 2019; Hipp et al., 2019). Key UPR molecules including BiP and PERK also become damaged over time and hinder the PN’s efficiency to respond to ER stress (Chadwick and Lajoie, 2019). Given that a hallmark of an aging proteome is a decline in protein solubility, this causes proteins to aggregate, making the PN work harder, and ultimately creating a positive feedback loop and further disturbs proteostasis (Hipp et al., 2019).

While our PN steadily declines with age, ER stress has been linked to multiple conditions, including diabetes mellitus, neurodegeneration, cancer, heart disease, and arrhythmias (Oakes and Papa, 2015; Liu and Dudley Jr., 2018; Chadwick and Lajoie, 2019). Mutations due to protein misfolding, aggregating proteins, and dysfunctional UPR sensors have been shown to lead to these conditions (Oakes and Papa, 2015). A declining PN introduces the risk of incorrect folding of insulin’s precursor, preproinsulin, which can lead to diabetes (Oakes and Papa, 2015; Chadwick and Lajoie, 2019). Misfolded proteins can be toxic once secreted from the ER and can harm neurons, which can lead to neurodegeneration (Oakes and Papa, 2015). In the case of cancer, the unfavorable conditions, such as hypoxia, nutrient deprivation, and oxidation, that trigger ER stress are often where tumor cells infiltrate and metastasize (Oakes and Papa, 2015). CMs near the myocardial infarct also experience hypoxia and ER stress, which initiates the UPR. However, since this tissue normally experiences chronic ER stress, terminal UPR signals the CMs toward a pro-apoptotic cell fate, leading to significant CM loss and potentially heart failure (Oakes and Papa, 2015).

The UPR is especially important for the maintenance of our heart, given CMs’ lack of regenerative capabilities (Liu and Dudley Jr., 2018). Since adult CMs cannot efficiently repair themselves or replicate, the existing cells must be carefully taken care of. The SR, a specialized domain of the ER, tightly regulates Ca^2+^ stores and transients within CMs that allow for the cell to survive and function as expected (Liu and Dudley Jr., 2018). Therefore, it is especially crucial that the ER is regulated and maintained within its functional limit to ensure CM viability and cardiac function. As mentioned previously, cardiovascular diseases put great stress on the ER due to the extreme conditions the CMs endure. Therefore, these conditions tend to trigger chronic ER stress, which can then further lead to cardiac dysfunction (Liu and Dudley, 2018).

## Inflammation in AF

**Figure 2:**
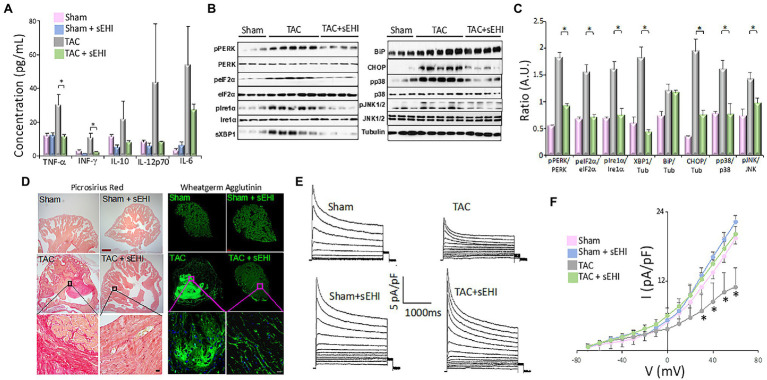
**(A)** Serum concentration of cytokines from Sham, Sham + sEHI, TAC, and TAC + sEHI treated mice. **(B)** ER Stress assay from Sham, TAC, and TAC + sEHI treated mice. **(C)** Bar graphs representing normalized data. **(D)** Cardiac sections stained with Picrosirius red and wheatgerm agglutinin showing an increase in collagen deposition in Sham, Sham + sEHI, TAC, and TAC + sEHI treated mice. Scale bar: red-500 μm and black-20 μm. **(E)** Transient outward K^+^ current recordings from single isolated atrial myocytes and **(F)** the corresponding current–voltage (I–V) plot from Sham, Sham + sEHI, TAC, and TAC + sEHI treated mice. *n* = 3–5 animals. ^*^p < 0.05 by ANOVA. Mean ± SEM (Sirish et al., 2016).

Inflammation is one of the major risk factors that has been linked to AF. Both local inflammation as seen in patients with myocarditis and systemic inflammation associated with post coronary artery bypass grafting or autoimmune diseases are correlated with increase AF risk (Morgera et al., 1992; Bruins et al., 1997; Lazzerini et al., 2016). Inflammatory infiltrates and increased serum levels of proinflammatory cytokines have been shown to be present in both animal models and patients with AF (Sun et al., 2007; Corradi et al., 2008; Harada et al., 2015). In our study using mouse pressure overload thoracic aortic constriction (TAC) model, we demonstrated a significant increase in proinflammatory cytokine and chemokine levels including interferon-γ (IFN-γ), TNF-α, and monocyte chemoattractant protein-1 (MCP-1) in the TAC mice compared to sham animals (Figure 2A; Sirish et al., 2016). We utilized an *in vitro* model of human induced pluripotent stem cell derived-cardiomyocytes (hiPSC-CMs) to elucidate the critical downstream NF-κB signaling cascade affected by the increase in inflammatory cytokine. Our data demonstrated an increased nuclear translocation of NF-κB in hiPSC-CMs and a significant increase in the pIκBα and nuclear NF-κB in response to TNF-α stimulation. Activated NF-κB increases the gene expression of inflammatory cytokines, intensifying inflammation and eventually contributing to atrial remodeling substantiating the “AF begets AF” phenomenon.

## Role of ER Stress in AF

Several studies have correlated inflammatory cytokines, such as MCP-1, with disruption protein folding in the ER leading to the activation of ER stress response (Azfer et al., 2006). ER stress has been shown to be involved in the pathophysiological basis of AF (Yuan et al., 2020). The ER in the heart contributes to the regulation of excitation-contraction coupling and perturbation in the ER homeostasis due to intrinsic and extrinsic factors, such as inflammation, oxidative stress, and ischemia, leads to ER stress, which contributes to cardiac hypertrophy, fibrosis, and apoptosis (Park et al., 2012; Groenendyk et al., 2013; Luo et al., 2015). Cardiomyocyte apoptosis further contributes to the development and maintenance of AF (Castillero et al., 2015). ER stress results in the activation of ER transmembrane protein sensors PERK and IRE1α, the upregulation of ER chaperones, such as BIP, initiation of ER-related apoptotic proteins, such as CHOP, and activation of mitogen-activated protein kinase (MAPK) signaling cascade (Groenendyk et al., 2013; Bettaieb et al., 2014; Harris et al., 2015). We have demonstrated that chronic pressure overload in TAC animals resulted in the activation of BIP, CHOP, PERK, and IRE1α, and their downstream targets α-subunit of eIF2α and XBP1. We also demonstrated a significant increase phosphorylation of PERK (Thr980), IRE1α (Ser724), p38 (Thr180/Tyr182), and c-Jun N-terminal kinases (JNK, Thr183/Tyr185) in atrial tissues contributing to increase in ER stress, which further promotes the production of inflammatory cytokines (Figures 2B,C; Sullivan et al., 2020).

ER stress can potentially be targeted to mitigate cardiac remodeling in AF. Wiersma et al. (2017) have elegantly shown that the use of chemical chaperone 4-phenyl butyrate (4PBA), an inhibitor of ER stress, prevents the activation of autophagy thereby, reducing electrical and contractile dysfunction in both *in vitro* and *in vivo* AF models. Using tachypaced HL-1 atrial CMs, they demonstrated that blocking ER stress using 4PBA or by overexpression of ER chaperone-protein heat shock protein A5 or mutant constructs of eIF2α prevents the activation of autophagy and Ca^2+^ transient loss. Similarly, pharmacological inhibition of ER stress and autophagy decreased dysfunction in heart wall contractions in tachypaced *Drosophila*. A large animal model of atrial-tachypaced mongrel dogs was used to demonstrate that inhibition of ER stress attenuated electrical remodeling, ER stress, autophagy, and AF progression.

## Experimental Models for the Studies of Atrial Arrhythmias

Experimental models to help elucidate atrial arrhythmogenesis and to investigate therapeutic potentials are profoundly impactful and significant for patients. Due to a variety of experimental models that currently exist for atrial arrhythmias and the differences in electrophysiology among the commonly used species (Milan and MacRae, 2005; Clauss et al., 2019), careful interpretations of the results are absolutely essential to translate findings to humans. Nonetheless, the array of experimental models available allows investigators to uncover mechanistic insights and to develop drug therapies to improve clinical outcomes.

## Cardiovascular Disease Models That May Induce Atrial Arrhythmia

Atrial arrhythmia is a common comorbidity with other cardiovascular complications and diseases, such as cardiac hypertrophy, myocardial infarction, ischemic and dilated cardiomyopathy, and HF (LaMori et al., 2013). Hence, animal models that have been used to induce cardiovascular diseases may be susceptible for AF. Commonly used models for cardiac hypertrophy and heart failure, such as aortic constriction, have been utilized in mice to study atrial structural and functional remodeling (Sirish et al., 2016). Indeed, other overload models, such as mitral regurgitation, mitral valve disease, and vascular shunt, have also been documented to induce atrial remodeling (De Jong et al., 2011), as structural and electrical remodeling of the atria contributes to the initiation and maintenance of AF (Polejaeva et al., 2016; Sirish et al., 2016). These surgical procedures produce atrial arrhythmias secondary to other cardiovascular diseases, which may be clinically relevant to study atrial arrhythmias in the context of other comorbidities, but may complicate atrial arrhythmogenesis investigation.

## Consideration for Animal Models

Animal models provide a wealth of knowledge to our current understanding of atrial arrhythmias in patients. However, careful interpretations of extrapolated results and understanding of each model’s limitations will provide more valuable insights. For instance, although small animals provide multiple benefits, such as the ease of breeding, inexpensive maintenance costs, and simplicity of genetic manipulation (Bryda, 2013), their cardiac anatomy and electrophysiology are largely different than humans (Boukens et al., 2014). Indeed, commonly used small rodents, such as mice, rats, and guinea pigs, exhibit shorter action potential (AP) duration (APD), primarily due to shortened and rapid repolarization phase (Edwards and Louch, 2017; Schüttler et al., 2020), which translate to a much higher heart rate relative to humans (Milani-Nejad and Janssen, 2014). In contrast, larger animals, such as rabbits, pigs, goats, and dogs, experience more similar APs as humans, but their generally small litter size, lengthy gestational period, ethical concerns, high maintenance cost, and difficulty for genetic modulation deter investigators from using them as their primary choice. Indeed, proof of concept is usually derived from smaller animals, which is further validated in larger animals, before it is applied to humans.

## Structural and Electrical Remodeling in AF

“Atrial cardiomyopathy” is a term that describes the pathological changes perpetuating AF and the key contributor of which is atrial fibrosis (Xintarakou et al., 2020). Atrial fibrosis is a complex process involving multiple contributors, including, excessive oxidative stress, pro-fibrotic cytokines, such as TNF-α, MCP-1, IL-6, IL-8, and ANG II, and their downstream mediators, MAPKs and TGF-β1 (Kupfahl et al., 2000; Burstein and Nattel, 2008; Sirish et al., 2013; Xintarakou et al., 2020). TGF-β promotes atrial fibrosis by activating Smad transcription factors, which activate the promoters of collagen I and III genes and by suppressing the activity of matrix metalloproteinases and protease inhibitors (Kupfahl et al., 2000; Dobaczewski et al., 2011; Sirish et al., 2013). Increased ROS production in CMs causes the activation of members of the MAPK pathway, extracellular signal regulated kinase 1 and 2 (ERK1/2) and JNKs, and the members of the TGF-β superfamily, all of which promote myocardial hypertrophy (Zhang et al., 2003; Dobaczewski et al., 2011; Bjornstad et al., 2012). We have demonstrated an increase in both atrial fibrosis using Picrosirius red stain and wheat germ agglutinin (Figure 2D) and atrial myocyte hypertrophy in the chronic pressure overload model. We also specifically demonstrated a significant increase in the percentages and the proliferative capacity of atrial fibroblasts from TAC mice, human atrial appendage as well as hiPSC-fibroblasts, and hiPSC-ACMs (atrial cardiomyocytes) in response to ANG II treatment. We also examined the activation of downstream members MAPK and TGF-β, the ERK1/2 and Smad2/3 in atrial fibroblasts, and myocytes. We demonstrated a significant elevation in the levels of phosphorylated ERK1/2 (pERK1/2) in atrial fibroblasts and myocytes in the TAC mice suggesting the activation of atrial fibroblasts, the leading contributor of adverse atrial structural remodeling associated with AF.

Abnormalities in electrical impulse formation or impulse conduction can initiate and maintain AF. Chronic or persistent AF alters the AP wavelength, which induces rapid AP rates in the atria, further feeding the arrhythmia (Nattel et al., 2008). While structural atrial remodeling hinders the propagation of AP therefore decreasing the conduction velocity, atrial electrical remodeling effects the refractory period, and the APD. The rapid atrial rate can be due to one of many possibilities. (1) The ectopic activity could be caused because of the slope of the phase 4 of the atrial AP is accelerated, which could be partially because of the increased atrial expression of the ion channels subunits of “funny current” (*I_f_*). (2) Another cause of rapid atrial rate delayed afterdepolarizations (DADs) can be attributed to the abnormalities in Ca^2+^ overload which causes the cell firing when the DAD becomes large enough to reach the threshold potential. (3) Early afterdepolarization (EADs): excessive prolongation of APD causes Ca^2+^ currents to recover from inactivation leading to early after depolarization and maintenance of AF (Wakili et al., 2011). Our *ex vivo* optical mapping of isolated atria demonstrated a slowing of activation, a prolonged APD in left atria, and importantly an increased dispersion of APD and effective refractory period, a known pro-arrhythmic factor. At the cellular basis, we show that electrical remodeling involves downregulation of transient outward K^+^ current (*I*_to_) in atrial cells which can significantly alter AP shape and duration contributing to electrical remodeling (Figures 2E,F). The reduction in *I*_to_ has been shown to be due to the decrease in the auxiliary subunit of the K^+^ channel thereby impairing the channel assembly *via* the activation of NF-κB signaling cascade (Panama et al., 2011).

We and others have shown the critical role of K^+^, Na^+^, and Ca^2+^ channel dysfunction in cardiac arrhythmias (Remme and Bezzina, 2010; Lu et al., 2015; Chen et al., 2016; Chiamvimonvat et al., 2017; Weisbrod, 2020). Genetic variations in genes of the pore forming subunits or accessory β-subunits of the rapid and slow delayed rectifier K^+^ channels (*I_Kr_* and *I_Ks_*) have been linked to human arrhythmia syndromes (Chen et al., 2016; Chiamvimonvat et al., 2017). Single-nucleotide polymorphisms in the ion channel genes including small conductance Ca^2+^-activated K^+^ channels (SK), K_v_11.1 (hERG) and the α-subunit of the Na_v_1.5 sodium channel (*SCN5A*) significantly increase AF susceptibility (Remme and Bezzina, 2010; Zhang et al., 2021). L-type calcium channel (Ca_v_1) remodeling has been shown to be important for AF in both mouse-models and in patients (Lu et al., 2015). The abnormal splice variants of *SCN5A* in the failing hearts trapped in the ER activate PERK, causing the downregulation of the full-length of normal Na_v_1.5 protein expression, resulting in the decrease in conduction velocity (Liu and Dudley Jr., 2018). Using specific inhibitors of PERK and IRE1 arm, Dudley’s group has elegantly shown that the UPR activation causes the downregulation of multiple cardiac ion channels, including Ca_v_1.2, hERG, and K_v_LQT1, resulting in APD prolongation and the reduction in the AP upstroke velocity (Liu and Dudley Jr., 2018). UPR directly and indirectly through UPR-induced oxidative stress, altered glycosylation, and Ca^2+^ homeostasis contributes to ion channel remodeling resulting in increased arrhythmic risk.

## Upstream Therapeutic Targets for Atrial Fibrillation

A recently publish study by Dudley’s group showed that the pharmacological inhibition of PERK reduced arrhythmia risk by altering ion channel regulation post-MI (Liu et al., 2021). Specifically, post-MI mice exhibited downregulation of ion channel proteins as well as ionic currents compared to the sham group. Ion channels affected by the PERK activation were identified by treating MI mice with PERK inhibitor and using cardiac-specific *PERK-KO* mice, leading to a significant improvement in channel availability, expression, and conduction velocity. However, reductions in Ca_v_1.2 and Kir2.1 were not rescued with the treatment, demonstrating that PERK directly influences only Na_v_1.5, K_v_1.5, and K_v_4.3 channels.

Inflammation has been implicated in the pathophysiology of AF through its effect on signaling pathways that lead to the development and maintenance of AF (Everett and Olgin, 2007; Burstein and Nattel, 2008; Guo et al., 2012). In addition, inflammation has been associated with comorbidities that predispose patients to AF (Guo et al., 2012; Harada et al., 2015). AF can aggravate inflammation which further perpetuates the arrhythmia. Hence, targeting inflammation has become the focus of new therapeutic strategies for the treatment of AF. One of the most biologically important groups of oxylipins is the eicosanoids, which are derived from the 20-carbon atom arachidonic acid. Tissue injury leads to the activation of phospholipase A_2_ and the release of arachidonic acid, which is metabolized through the cyclooxygenase (COX), lipoxygenase (LOX), and cytochrome P450 (CYP450) pathways. While several of the COX and LOX metabolites are proinflammatory and have been studied in detail, underpinned by the translation of inhibitors of these enzymatic pathways demonstrated by aspirin and zileuton in the treatment of inflammatory diseases, the translational manipulation of the CYP450 pathway remains unexplored and underutilized clinically. The CYP450 epoxidized products, the epoxyeicosatrienoic acids (EETs) have been shown to have anti-inflammatory with several cardioprotective effects (Li et al., 2009, 2011; Liu et al., 2010; Qiu et al., 2011; Sirish et al., 2013, 2020). EETs function primarily as autocrine and paracrine effectors in the cardiovascular system and kidney (Roman et al., 2000; Schmelzer et al., 2005). EETs modulate ion transport and gene expression, producing vasorelaxation, anti-inflammatory, and pro-fibrinolytic effects. All EET regioisomers function as endogenous hypotensive agents (Katoh et al., 1991; Roman et al., 2000). However, EETs are further metabolized by soluble epoxide hydrolase (sEH) to form the corresponding diols (DHETs) with diminished anti-hypertensive and anti-inflammatory activities and we and others have found that there is a significant decrease in EETs/DHETs ratios in several diseased models (Zeldin et al., 1996; Yu et al., 2000). To increase the cardioprotective activity of endogenous EETs, novel inhibitors of sEH (sEHI) can be used to block the degradation of EETs to corresponding DHETs (Morisseau and Hammock, 2005).

We have previously demonstrated the beneficial effects of sEHIs in clinically relevant models of cardiac hypertrophy and failure, resulting in a significant improvement in cardiac function (Xu et al., 2006; Li et al., 2009, 2011; Sirish et al., 2013). We further demonstrated that treatment with sEHIs results in the prevention of ventricular myocyte hypertrophy, electrical remodeling, cardiac fibrosis, and reduces both atrial and ventricular arrhythmia inducibility in MI models (Chiamvimonvat et al., 2007; Li et al., 2009, 2011; Sirish et al., 2013, 2016). Metabolic profiling was utilized to unravel one of the molecular mechanisms underlying the prevention of arrhythmia inducibility and electrical remodeling with sEHI treatment, which is the normalization of *I*_to_ downregulation, which is well described in AF (Li et al., 2009; Sirish et al., 2013, 2016). Our study with the pressure overload model, which represents the development of hypertension, a major risk factor for AF, shows a decrease in the EETs/DHETs ratio in the TAC animals, which was increased with sEHI treatment (Sirish et al., 2016). Moreover, analysis of arachidonic acid metabolites of the *COX* pathway demonstrates an increase in proinflammatory thromboxane and prostaglandin levels in the TAC model which was attenuated with sEHI treatment (Sirish et al., 2016). Thus, we demonstrated that treatment with sEHIs to normalize the EETs/DHETs ratios represents an unexplored avenue to modify atrial fibrosis, alleviate inflammatory cytokines and chemokines, and reduce atrial electrical remodeling, all of which help in the prevention and progression of AF. Very little is known about the role of this new class of compounds in the treatment of AF. There is consequently an enormous opportunity to uncover a potentially very powerful class of compounds, which may be used effectively in the clinical setting.

## Author Contributions

PS, DD, PT, and NC performed the literature searches and prepared the manuscript. PS and DD generated the figures. All authors contributed to the article and approved the submitted version.

## Funding

This study was supported in part by Postdoctoral Fellowship from the NIH/NHLBI Institutional Training grants NIH T32 HL086350 and NIH F32 HL149288 (PT), NIH R01 HL085727, NIH R01 HL085844, and NIH R01 HL137228 (NC), VA Merit Review grant I01 BX000576 and I01 CX001490 (NC), and AHA Postdoctoral Fellowship Award, Harold S. Geneen Charitable Trust Award Program for Coronary Heart Disease Research, AHA Career Development Award, 18CDA34110060 (PS).

## Conflict of Interest

The authors declare that the research was conducted in the absence of any commercial or financial relationships that could be construed as a potential conflict of interest.

## Publisher’s Note

All claims expressed in this article are solely those of the authors and do not necessarily represent those of their affiliated organizations, or those of the publisher, the editors and the reviewers. Any product that may be evaluated in this article, or claim that may be made by its manufacturer, is not guaranteed or endorsed by the publisher.
